# Hsp70 at the membrane: driving protein translocation

**DOI:** 10.1186/s12915-017-0474-3

**Published:** 2018-01-17

**Authors:** Elizabeth A. Craig

**Affiliations:** Department of Biochemistry, University of Wisconsin - Madison, 433 Babcock Drive, Madison, WI 53706 USA

## Abstract

Efficient movement of proteins across membranes is required for cell health. The translocation process is particularly challenging when the channel in the membrane through which proteins must pass is narrow—such as those in the membranes of the endoplasmic reticulum and mitochondria. Hsp70 molecular chaperones play roles on both sides of these membranes, ensuring efficient translocation of proteins synthesized on cytosolic ribosomes into the interior of these organelles. The “import motor” in the mitochondrial matrix, which is essential for driving the movement of proteins across the mitochondrial inner membrane, is arguably the most complex Hsp70-based system in the cell.

## Challenges in protein translocation across membranes

Proteins synthesized on cytosolic ribosomes and translocated across membranes into organelles play critical roles in cell and organismal physiology. Translocation of proteins into the endoplasmic reticulum (ER) and mitochondria is especially demanding. The protein complexes embedded in the membrane, referred to as translocases or translocons, through which the proteins must pass, have narrow channels [1, 2]. They are able to accommodate only a completely unfolded chain or, at most, an α-helix. Thus, postponing folding, yet preventing aggregation, of a protein is necessary for its efficient translocation. In addition, protein movement must not only be vectorial, that is, unidirectional from the cytosol into the organelle, it must also be efficient to keep up with the heavy cellular demand for organelle function.

For many ER proteins, the co-translational nature of the translocation process overcomes such hurdles. Coupling of protein translation and protein translocation minimizes the issue of tertiary structure hindering passage through the translocation channel, while using the “force” of protein synthesis to drive directional movement across the membrane. Via action of signal recognition particle (SRP) binding to targeting sequences at the N-terminus of an ER-destined protein, the translating ribosome docks directly onto the translocon of the ER membrane [3, 4]. This precise docking provides a direct conduit for the nascent polypeptide chain from the ribosome exit tunnel through the channel in the membrane-imbedded translocon [1]. However, in organisms as diverse as budding yeast (S*accharomyces cerevisiae*) and humans, a substantial number of proteins are translocated post-translationally into the ER [5]. Moreover, mitochondria have no exact analog of the SRP system that results in a direct physical connection between the ribosome and the translocon of the outer mitochondrial membrane.

Hsp70 molecular chaperones function both in the cytosol and internally on the luminal/matrix face of ER/mitochondrial membranes, helping cells overcome these inherent challenges of protein translocation across membranes. In this review, particular attention is given to the Hsp70 system of the mitochondrial matrix, which is required for the translocation of all nuclear-encoded proteins into this subcompartment [6]. Many fundamental aspects of both ER and mitochondrial translocation systems have been highly conserved in evolution. Throughout, *S. cerevisiae* nomenclature is used as much of the work to understand the mechanism of protein import and molecular chaperone function was performed using this model organism.

## Properties of Hsp70s critical for cellular functions

Hsp70 molecular chaperones are present in all major cellular compartments (i.e., cytosol, nucleus, ER, and mitochondria), functioning in diverse cellular processes from protein folding to disassembly of protein complexes to protein translocation across membranes. While the protein translocation is the focus of this review, Hsp70s, when involved in any of these processes, bind to seven-residue segments of polypeptide that are overall hydrophobic in nature [7]. Virtually every protein that is not folded into its native state has multiple accessible Hsp70 binding sites, because residues found in the hydrophobic core in the native conformation are exposed. It has been estimated that most proteins have an Hsp70 binding site every 30–40 residues [8].

**Fig. 1.**
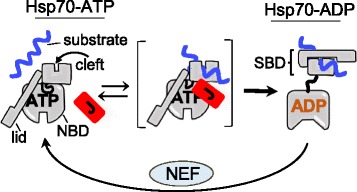
Overview of the Hsp70–substrate interaction cycle. Kinetics of the Hsp70–substrate interaction cycle are driven by ATP binding and hydrolysis, followed by exchange of ADP for ATP. Hsp70s have two domains: a nucleotide binding domain (*NBD*) and a substrate binding domain (*SBD*). The SBD has two subdomains; one has the substrate binding cleft, the other the lid, which can cover the cleft, trapping substrate (*right*). When ATP is bound (*left*), Hsp70 is in what is called the open- or docked-state. Substrate has easy access to the substrate binding cleft in the SBD because both subdomains of the SBD are restrained by interaction with the NBD. Although this conformation allows a high on-rate of substrate binding, the off-rate is also rapid. Binding of the J-domain (*J*) of a J-protein co-chaperone at the NBD–SBD interface, in concert with substrate in the cleft, stimulates hydrolysis of ATP to ADP. The resulting conformational changes cause the domains to disengage, forming the undocked/closed state and stabilizing substrate interaction by closure of the lid over the cleft. The *brackets* indicate dynamic transitions between the predominant ATP and ADP conformations. Nucleotide release by nucleotide exchange factors (*NEF*) and rebinding of ATP completes the cycle

Cycles of interaction with substrate polypeptides is an important aspect of Hsp70 function, not only in protein translocation, but in other functions such as protein folding and disassembly of protein complexes. Hsp70–substrate interactions are controlled by ATP binding and hydrolysis (Fig. 1) [9]. When ATP is bound to Hsp70, the substrate on-rate is very rapid, but so is the off-rate. ATP-hydrolysis results in trapping the substrate polypeptide, and nucleotide exchange results in rapid dissociation. Two types of co-chaperones regulate the Hsp70–substrate interaction cycle [10]. One, J-proteins, via the action of their highly conserved J-domain, stimulates ATP hydrolysis and thus stabilization of substrate interaction. The other, nucleotide exchange factors (NEFs), drives exchange of ADP for ATP, facilitating substrate release.

**Fig. 2.**
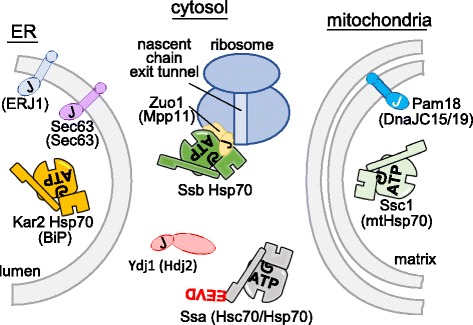
Hsp70s involved in protein translocation across the endoplasmic reticulum (*ER*) and mitochondrial membranes. Hsp70s and J-proteins of *Saccharomyces cerevisiae* are indicated, with the commonly used names for the orthologous proteins in human cells in parentheses. J-domains indicated by “*J*”. The major class of Hsp70 found in the cytosol of all eukaryotes is called Ssa or Hsc70/Hsp70 in fungi and other eukaryotes, respectively. An EEVD tetrapeptide present at the C-terminus targets these Hsp70s to interacting proteins such as receptors on organelle membranes, Hsp90 molecular chaperones, or proteolytic systems. These Hsp70s are encoded by between one and four genes in fungi, depending on the species, and by five genes (HSPA1, 2, 6, 7, 8) in humans. Fungi have another type of cytosolic Hsp70, called Ssb, which is predominately ribosome-associated. Humans have no Ssb ortholog; rather, the Hsc70/Hsp70 type performs the equivalent functions. Both Ssb and Hsp70/Hsc70 partner with a conserved, specialized J-protein, Zuo1 in fungi and Mpp11 (DNAJC2) in other eukaryotes, which is predominately ribosome-associated. Ydj1 (Hdj2 or DNAJA1 in other eukaryotes) is the most abundant J-protein partner of those cytosolic Hsp70s involved in protein translocation. In most eukaryotes, the lumen of the ER and the mitochondrial matrix have a single Hsp70, which plays multiple roles in their respective organelles, including general protein folding. ER Hsp70 is often called BiP; encoded by *KAR2* (fungi) or *HSPA5* (humans). Mitochondrial Hsp70 (mtHsp70) is called Ssc1 in fungi. In humans mtHsp70 is encoded by *HSPA9*. Some fungi have an additional Hsp70 (called Ssq1) specializing in Fe-S cluster biogenesis, and a low abundance Hsp70, Ecm10. Sec63 (ERdj2) in human cells is encoded by DNAJC23. ERJ1 (ERdj1) is not present in fungi. Pam18 (also called Tim14) of fungi has two orthologs in human cells, DNAJC17 (or Tim14) and DnajC15, sometimes called MCJ

Although these fundamental principles apply to all Hsp70 systems, specialization is common. An Hsp70 typically has multiple different J-protein partners, which may either target Hsp70 to a particular site within a compartment or bind a substrate itself, targeting it to the Hsp70 [11]. For example, the single Hsp70 of the ER partners with six J-proteins. Also, Hsp70s themselves may have specialized interactions, independent of their substrate binding, that render them more effective in specific cellular roles, including protein translocation. For example, the major cytosolic Hsp70s of all eukaryotes (called Ssa in *S. cerevisiae* and Hsc70/Hsp70 in metazoans) have a conserved EEVD tetrapeptide at their C-terminus, serving to target them to particular binding partners [12], including receptors at the membrane, as described below. In addition, although most metazoans have only the Hsc70/Hsp70 type of Hsp70 in the cytosol, fungi have a second type, called Ssb [13, 14]. Both Ssa and Ssb Hsp70s are involved in protein translocation across membranes (Fig. 2).

## Routes to the mitochondria and ER involving Hsp70 action

**Fig. 3.**
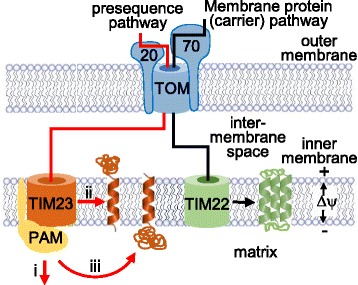
Pathways to the mitochondrial matrix and inner membrane. All proteins destined for the matrix or inner membrane cross the outer membrane through the TOM translocon (*blue*). Two receptor proteins, Tom20 (20) and Tom70 (70), which are part of the TOM complex, engage these proteins on the cytosolic side of the outer membrane. These proteins then use one of the two translocases present in the mitochondrial inner membrane: TIM22 (*green*) and TIM23 (*brown*). Proteins that are particularly hydrophobic, such as metabolite carrier integral membrane proteins, typically use the Tom70 receptor, then the TIM22 translocon, from which they are laterally transferred into the inner membrane (*black arrow*). Proteins with N-terminal cleavable targeting sequences typically use the presequence pathway: the Tom20 receptor, then the TIM23 translocon (*red arrow*). The membrane potential across the inner membrane (*∆ψ*) drives the positively charged presequence across the membrane. Three routes of proteins utilizing the TIM23 translocon: (*i*) the presequence associated motor (*PAM*) drives the remainder of the protein in the matrix; (*ii*) proteins with “stop transfer” sequences move laterally into the inner membrane; (*iii*) proteins with multiple domains may be partially imported by PAM and then move laterally into the membrane via the signal of an internal stop-transfer sequence

To reach their destination, ER and mitochondrial proteins utilize a variety of translocation pathways. For example, all nuclear-encoded proteins destined for the mitochondrial matrix pass through two translocons, the TOM complex of the outer membrane and the TIM23 complex of the inner membrane [2] (Fig. 3). Integral mitochondrial inner membrane proteins, which also utilize the TOM translocon of the outer membrane, are laterally transferred into the inner membrane via one of two inner membrane translocases: the TIM22 translocon, which is dedicated to integral membrane proteins, or the TIM23 translocon. The TIM23 route is often called the “presequence pathway”, because the proteins utilizing this pathway, whether they end up wholly within the matrix or in the inner membrane, are typically synthesized with N-terminal targeting sequences (presequences) that are cleaved in the matrix. As discussed below, the TIM22 pathway is particularly dependent on cytosolic Hsp70s, while the translocation of proteins into the matrix via the presequence (TIM23) pathway requires matrix Hsp70 activity. To reach the ER lumen, many proteins utilize the SRP pathway through the SEC61 translocon of the ER membrane. This route does not require Hsp70 action (Fig. 3). However, some, particularly short lumenal polypeptides or those with less effective targeting sequences often do not bind SRP, but rather are translocated post-translationally through SEC61, relying heavily on cytosolic Hsp70 (and also lumenal Hsp70; see below).

## Hsp70 on the cytosolic side of the membrane

Data obtained in the 1990s pointed to a role for cytosolic Hsp70 chaperones in the translocation of proteins into both the ER and mitochondria [15–18]. The idea put forth at the time, and consistent with emerging in vitro evidence that chaperones help prevent aggregation of unfolded proteins [19], was that binding prevents formation of tertiary structure that hinders threading of the protein through narrow translocation channels. Overall, work over the ensuing years has supported this general idea. Recent data have brought both clarification and evidence of unanticipated complexity. On one hand, the issue of aggregation of proteins destined for the mitochondria is likely not as extreme as originally envisioned. Translation of many proteins destined for mitochondria is now known to occur at the mitochondrial outer membrane, in close proximity to the TOM complex [20, 21], rather than in the bulk cytosol, as previously thought. On the other hand, post-translational translocation into the ER via mechanisms not dependent on SRP are more common than previously appreciated [22]. As described below, besides helping to maintain proteins in a partially folded, yet soluble state, Hsp70 binding targets substrates to the mitochondrial outer membrane and ER translocon channels. In both cases the C-terminal EEVD tetrapeptide of Hsp70 is involved in the targeting.

**Fig. 4.**
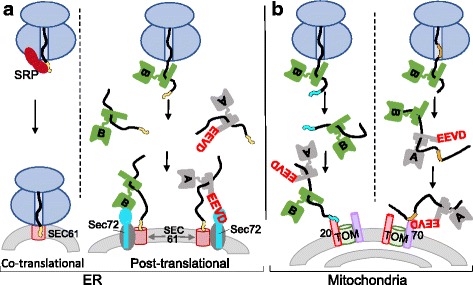
Hsp70 in protein translocation on the cytosolic side of the membrane. **a**, **b** *Top*: Nascent polypeptides (*black line*) travel through the tunnel of the ribosome (*blue*), before exiting into the cytosol. **a** Many polypeptides destined for the endoplasmic reticulum (*ER*) are synthesized with a targeting sequence at or near the N-terminus (*yellow line segment*). *Left*: Co-translational translocation. Signal recognition particle (*SRP*) binds targeting sequence (*yellow*), halting translation and targeting the nascent chain to SRP receptor (not shown), then to SEC61 translocon, at which point translation resumes. *Right*: Post-translational translocation. Nascent polypeptides with signal sequences not recognized efficiently by SRP are bound by Hsp70 Ssb (*green*, “*B*”) that is associated with ribosomes at the tunnel exit and/or soluble Hsp70 Ssa (*gray*, “*A*”). The Ssa C-terminal EEVD tetrapeptide is in *red*. Ssa and Ssb target nascent chains to SEC61 by binding to Sec72, a component of the Sec62/63 complex (Sec62/63, *dark gray*). Ssa interacts via its C-terminal EEVD tetrapeptide, and Ssb via its nucleotide binding domain. Not shown: J-proteins needed for Hsp70 binding to polypeptide substrate to facilitate ATP hydrolysis and NEF for exchange of nucleotide and thus release of substrate from Hsp70. **b** Mitochondria: Tom20 (*pink cylinder*) and Tom70 (*purple cylinder*), components of the TOM complex embedded in the outer membrane, are receptors for proteins destined for the inner membrane and matrix. *Left*: Proteins that bind Tom20 typically have an N-terminal, cleavable targeting sequence (*cyan line segment*). *Right*: Tom70 targeting sequences (*orange line segment*) are typically in the protein’s interior. Tom70 also binds the EEVD of Ssa type Hsp70s, helping to target these polypeptides to the TOM translocon

The outer mitochondrial membrane TOM complex is composed of channel-forming Tom40 and associated proteins, including the two receptor proteins Tom20 and Tom70 (Fig. 4). Tom70 is the primary receptor for proteins that have internal hydrophobic targeting sequences, such as the abundant, integral inner membrane carrier proteins that utilize the TIM22 inner membrane translocon (e.g., ATP/ADP carrier) [23, 24]. In addition, Tom70 binds Hsp70’s C-terminal EEVD via its tetratricopeptide repeat (TPR) domain [25]. This dual interaction is likely regulatory, with conformational changes upon EEVD binding linking receptor activation to chaperone binding. Tom20, the primary receptor for the presequence pathway, does not have an EEVD binding site. Perhaps the challenge of preventing aggregation of abundant integral membrane proteins was behind evolution of direct chaperone–receptor interactions. In mammalian cells, but not yeast cells, the molecular chaperone Hsp90 acts similarly, interacting with the TPR domain of the Tom70 receptor through its conserved C-terminal EEVD [26, 27].

During post-translational translocation into the ER, the EEVD of Ssa Hsp70 interacts with the TPR domain of Sec72, a SEC61 translocon-associated protein [28] (Fig. 4). Though lacking a C-terminal EEVD tetrapeptide, Ssb Hsp70s, which associate with ribosomes near the exit of the tunnel, also interact with Sec72 [28]. This interaction occurs via Ssb’s N-terminal nucleotide binding domain. Metazoans do not have a Sec72 homolog, but mammals have a second J-protein, ERj1 (DnaJC1) in the ER membrane. The lumenal J-domain functions in protein translocation across the membrane. A cytosolic domain binds ribosomes near the tunnel exit site [29], and may help recruit them to the ER membrane.

Analysis of individual proteins gives substantial support to the idea that the Ssa Hsp70 class plays a significant role in post-translational translocation [30–33]. For Ssb Hsp70s, recent in vivo selective ribosome profiling data provide genome-wide insights into the breadth of its nascent chain interactions [34]. On the order of 80% of the different nascent chains known to be destined for mitochondria were found to bind to Ssb, consistent with observed aggregation of mitochondrial proteins in cells lacking Ssb [35] and the ability of increased expression of Ssb to overcome the growth defect caused by inefficient mitochondrial protein translocation [36]. Ssb also interacts with almost half of all the different ER proteins. While most of these interactors do not require SRP for ER targeting (e.g., tail anchored proteins [37]), Ssb also binds a significant number of proteins known to transit into the ER via the SRP-dependent mechanism. In these, the first Ssb binding site to emerge from the ribosome is typically more N-terminal than the SRP binding site [34]. Many questions remain. Does an individual nascent chain bind both Ssb and SRP? Is there a mechanistic cooperation between Ssb and SRP, perhaps a handing-over from one system to the other? Or is this binding indicative of alternative pathways, SRP-dependent and SRP-independent?

## Hsp70 on the matrix/lumenal side of the membrane

Hsp70s in the mitochondrial matrix and the ER lumen play a critical and more active role in protein translocation than do those on the cytosolic side. They form the core of the machinery, often called “import motors”, that binds the translocating polypeptide and drives it across the membrane. Hsp70s of both these import motors utilize the same biochemical properties to drive translocation as used by Hsp70s when functioning in other biological processes—initial interaction of Hsp70-ATP with substrate, stabilized by J-protein driven ATP hydrolysis, then destabilized by NEF-driven nucleotide exchange. ER lumenal Hsp70 (officially Kar2 in yeast; but often called BiP in both yeast and metazoans) drives post-translational import of proteins through the Sec61 channel [1, 5]. Sec63 is this motor’s dedicated J-protein; it associates with the SEC61 complex, as a component of the Sec62/63 complex, which in yeast also includes Sec71/72. PAM, the mitochondrial presequence associated motor of the matrix, provides the driving force for movement of all nuclear-encoded matrix proteins [2, 6, 38]. Below I concentrate on mitochondrial PAM, as it has been studied much more extensively than the lumenal ER Hsp70 system.

### Steps of the presequence import pathway into the mitochondrial matrix

**Fig. 5.**
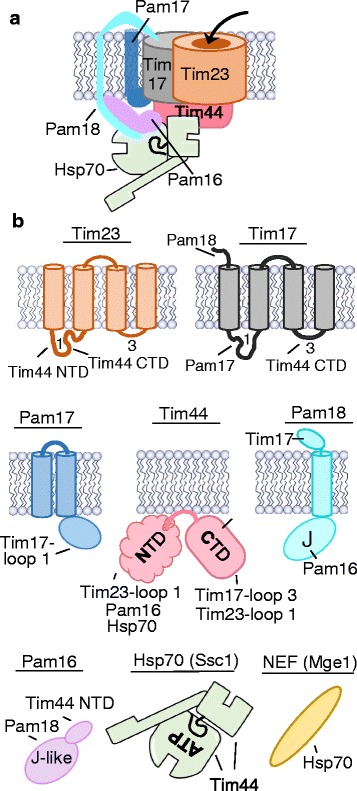
The presequence associated motor (PAM). **a** PAM architecture. Hsp70 is in its ATP-bound state, with cleft easily accessible for binding an incoming polypeptide that would enter through the Tim23 channel, as indicated by the *arrow*. Tim50, an essential component of the TIM23 translocon with an essential inter membrane space (IMS) domain, is not shown. Likewise, the N-terminal IMS-localized domain of Tim23 is not shown. Both interact with presequence prior to its entering the channel. The nucleotide exchange factor (NEF) Mge1 is not shown; it interacts with ADP-bound Hsp70, not the ATP-bound form that is present at the translocon. **b** Components shown in **a** are depicted individually. Interactions to particular domains observed by biochemical, structural, or site-specific crosslinking experiments are indicated with a *dash*. Matrix exposed loops of Tim17 and Tim23 are indicated by number (“*1*” and “*3*”). The N-terminal and C-terminal domains (*NTD* and *CTD*, respectively) of Tim44 are shown; NTD is represented as a “cloud” to indicate that it is intrinsically disordered

Before PAM can act, the N-terminus of the preprotein must enter the matrix. The N-terminal targeting presequence, an antipathic α-helix, interacts with a series of receptors as it moves from the cytosolic surface of the outer membrane to Tim23 complex in the intermembrane space [6]. It first interacts with the Tom20 receptor, then other components of the TOM complex, then components of the TIM23 complex. The membrane potential, negative on the matrix side, drives the positively charged presequence across the membrane. Translocation of the remainder of the polypeptide requires the action of the import motor, PAM (Fig. 5a).

The TIM23 complex contains three essential proteins. Two, Tim23 and Tim17, are related integral membrane proteins (Fig. 5b). Tim23 forms the channel; Tim17 likely plays a role in maintaining the translocon’s structure and channel gating [39–41]. Both have four transmembrane helices, with the N- and C-termini extending into the intermembrane space. Two loops (1 and 3) between the membrane spanning segments, on the order of 23 and 10 residues, respectively, extend into the matrix and serve as interaction sites for PAM. More than one molecule of both Tim23 and Tim17 are present in each TIM23 complex; the exact number is not known, a complicating factor in understanding the mechanism of action of the import motor. The third essential subunit is Tim50. Both Tim50 and Tim23 have domains that extend into the intermembrane space and interact with the targeting sequence, promoting the first step of translocation across the inner membrane, that is, opening of the gated Tim23 channel [42, 43].

The TIM23 complex is also responsible for the transport of some inner membrane proteins (Fig. 2). For many of these, PAM is not involved. Rather, the membrane potential drives the presequence targeting sequence through the channel; an adjacent “stop-transfer” sequence arrests movement and facilitates lateral transfer into the membrane [6, 44]. But in other cases, proteins are first imported into the matrix, then insertion into the inner membrane is facilitated by the action of the protein insertion machinery, called the oxidase assembly (OXA) system [45]. This process is often referred to as conservative sorting because of its resemblance to transport systems of bacteria, the progenitor of mitochondria [46]. In a few cases, the PAM/OXA system is used for some of a protein’s transmembrane domains, but the stop-transfer, lateral gating system for others [47, 48].

### Architecture of the presequence associated motor PAM

PAM is composed of six subunits (Fig. 5), five of which are essential. Three are core Hsp70 system essential components—Hsp70 Ssc1, J-protein Pam18 (also called Tim14), and NEF Mge1 [6, 38]. The Hsp70 and NEF are the same molecules that carry out other processes in the mitochondrial matrix, including general protein folding and remodeling of protein complexes [49]. However, J-protein Pam18, like its analog Sec63 in the ER lumen, is specific for protein translocation. When Ssc1 and Mge1 engage in other biological processes, they work with a different J-protein, such as Mdj1 [50, 51]. The two other essential components are Pam16 (also called Tim16) [52–54] and Tim44. Tim44 is considered the “hub” of the motor. It serves as the connector between the motor and the translocon, interacting with the TIM23 complex and with other motor components [55, 56]. The nonessential motor component Pam17, a membrane protein having a matrix domain, appears to play a yet-to-be clearly defined early role [57–59].

The architecture of PAM is complex (Fig. 5). Pam18 and Pam16, in addition to Tim44, have multiple interactions that provide functional redundancy and robustness. J-protein Pam18 has a single transmembrane segment. On the intermembrane space side of the membrane it interacts with Tim17 [60], and on the matrix side with Pam16 [61–64]. In turn, Pam16, via its N-terminus, interacts with Tim44 [63, 65]. Pam16 has a degenerate J-like domain, incapable of stimulating Hsp70’s ATPase activity. Rather, along with adjacent residues, the J-like domain interacts with Pam18’s J-domain [61, 62]. These interactions on both sides of the membrane are important for Pam18’s association with the TIM23 translocon [65].

**Fig. 6.**
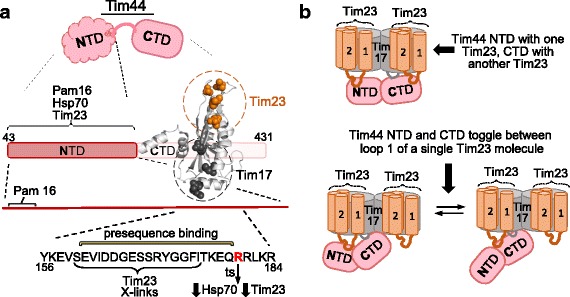
Tim44 and its interaction with the TIM23 translocon. **a** The multiple interactions of the two domains of Tim44: N-terminal domain (*NTD*) and C-terminal domain (*CTD*). In the expanded region of NTD, regions of Pam16 interaction, Tim23 crosslinking, presequence binding, and residue R180, the site of amino acid substitution (ts) that affects interaction of both Tim23 and Hsp70, are indicated. This “hot spot” is a candidate for an important role in Tim44 function, such as initiation activation of motor upon entrance of presequence into the matrix driven by the membrane potential. In the CTD (PDB entry 2FXT) residues at positions that crosslink, when having a photoactivatable amino acid, are shown in sphere representation crosslinked to Tim17 (*gray*) or Tim23 (*orange*). **b** Cartoon of Tim44 interaction with the TIM23 translocon, illustrating the dilemma posed by data indicating that both the NTD and CTD of Tim44 (*pink*) interact with 24-residue loop 1 of Tim23. This short length likely precludes simultaneous binding due to steric hindrance. *Top*: As the TIM23 translocon contains at least two Tim23 molecules, one Tim44 could interact with two Tim23 molecules simultaneously. *Bottom*: Alternatively, the NTD and CTD of Tim44 could toggle back and forth, interacting only with one Tim23 molecule, potentially playing a role in regulating or driving efficiency of the motor. Transmembrane helix 1 and 2 that flank loop 1 of Tim23 are indicated

The hub protein Tim44, a peripheral membrane protein, has two domains of approximately equal size (Figs. 5 and 6). The N-terminal domain (NTD) is intrinsically disordered [66]; the C-terminal domain (CTD) forms an α + β barrel with two N-terminal α-helices protruding from the core [67], which are thought to be involved in membrane association [68]. The NTD serves as the site of binding for both Hsp70 and Pam16 [65, 69]. Pam16 interacts with a small segment near the N-terminus [65]. Interaction with Hsp70 is likely more dispersed over the NTD, as both Hsp70 domains are involved in the Hsp70–Tim44 interaction [70–73]. The primary role of Tim44’s CTD is to interact with the TIM23 complex. Site-specific crosslinking indicates that adjacent patches on a face of the barrel interact with the TIM23 complex—one with Tim17 and one with Tim23 (Fig. 6). The large matrix-exposed loop of Tim17 (loop 1) interacts with one CTD patch and the small loop (loop 3) of Tim17 with the other [66, 74]. Loop 1 of Tim17 crosslinks to Pam17, the nonessential PAM component [75].

But the Tim44 picture is not as “simple” as the NTD interacting with the motor and the CTD with the translocon. Loop 1 of Tim23 also crosslinks to Tim44’s NTD [75]. This complex crosslinking pattern is consistent with the ability of the two domains to support viability when expressed separately (i.e., in *trans*) [76]; but such yeast cells grow very slowly, underscoring the complexity of Tim44 function. However, as Tim23’s loop 1 is only 24 residues, it is unlikely that both domains interact with the same Tim23 molecule simultaneously [66]. Thus, whether one Tim44 molecule interacts with two different Tim23 molecules simultaneously or interactions of the CTD and NTD occur sequentially to one Tim23 molecule remains an open question, leaving unresolved important mechanistic and regulatory questions raised below. That the stoichiometry of Tim23 in the TIM23 complex is also unresolved and that Tim44 has been reported to be a dimer further confound a mechanistic understanding of PAM function [56].

### How does an import motor mechanistically drive efficient protein translocation?

For many years two challenging questions have vexed workers studying PAM: what mechanistic principle(s) are behind motor function, and what motor characteristics drive motor efficiency? Two models of import motor action were put forward soon after it became clear that Hsp70 was required for post-translational translocation across membranes—“brownian (molecular) ratchet” and “power stroke” [77–79]. The mechanism by which Hsp70 binding to the translocating polypeptide drives directional movement is the fundamental difference between the two models. In the power stroke model, the polypeptide chain is pulled into the matrix by Hsp70 acting as a lever arm to generate force through conformational change, with Tim44 serving as a fulcrum. In the simplest form of the Brownian motion model, binding of Hsp70 to the translocating polypeptide prevents its backsliding because of its large size compared to the narrow import channel. Each model was appealing, yet problematic, in its own way. The power stroke model, as envisioned, helped rationalize data showing that Hsp70 binding not only drove translocation of an unfolded polypeptide into the matrix, but generated sufficient power to unfold a protein domain “stuck” at the outer membrane [80–82]. But whether Hsp70’s conformational changes are of sufficient magnitude to move the chain through the channel has not been critically addressed. On the other hand, the simplicity of the ratchet model was appealing. Indeed, early studies using an in vitro ER system showed Hsp70 BiP and J-protein Sec63 to be sufficient to move preproalpha factor, a small protein that is efficiently translocated post-translationally in vivo, through the SEC61 translocon [83]. However, it was difficult to envision how simply preventing backsliding would suffice energetically for more challenging substrates.

**Fig. 7.**
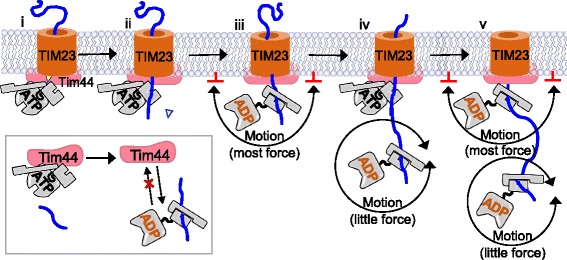
Model of Pam motor action. A model based on entropic pulling, an extension of the Brownian motion model, is shown. TIM23 translocon in inner membrane (*brown*); translocating polypeptide (*blue*); Tim44 (*pink*); Hsp70 (*gray*). (*i*) The presequence (*yellow*), upon entrance into the matrix driven by the membrane potential, binds Tim44’s NTD, perhaps activating the motor. (*ii*) Preprotein binds in the cleft of an Hsp70, which is tethered very close to the channel exit by Tim44. (*iii*) This binding, in conjunction with Pam18’s J-domain (not shown), stimulates Hsp70’s ATPase activity. The conformational change results in trapping of the translocating polypeptide and (see *insert*) release of Hsp70 from Tim44. According to the “entropic pulling” model a force is exerted because Hsp70’s movement is restricted by the translocon and membrane (indicated by *red bars*). (*iv*) As the translocating polypeptide, with Hsp70 bound, moves away from the membrane, the force is reduced because Hsp70’s motion is no longer restricted. Another Hsp70-ATP is able to bind Tim44, starting (*v*) another cycle of “directed” movement

An extension of the Brownian motion model, grounded in the more thorough consideration of the effects of binding of a large molecule such as Hsp70 to a translocating polypeptide close to the channel, has been developed [84]. According to this “entropic pulling” model, binding of Hsp70 at the exit pore generates a force, because “simple” restriction of its movement—“bumping into” the membrane or translocon—generates energy (i.e., a pulling force) (Fig. 7). The appeal of this model is that “simply” binding Hsp70 could generate a force without the need for either a fulcrum or a conformational change of a magnitude required to drive translocation at a biologically reasonable rate. Rather, “just” cycles of binding of Hsp70s to the incoming polypeptide could be sufficient. Similar force generation considerations arise when considering Hsp70 functioning in remodeling of protein complexes and dissolution of protein aggregates. Recent observation of uncoating of clathrin cages by Hsp70 and the J-protein auxilin are consistent with an entropic pulling model [85]. The juxtaposition of the auxilin and Hsp70 binding sites were critical; when moved further apart, the efficiency of the uncoating reaction decreased significantly. In addition, when an immunoglobulin binding site was placed at an appropriate position, addition of immunoglobulin alone facilitates cage disassembly.

For polypeptide translocation, the issues are more complex than uncoating clathrin cages. Not only must Hsp70 binding occur very close to the channel, but also a series of Hsp70 molecules must interact in rapid succession, each as close to the channel as possible. Interactions of Tim44 with motor components and with the translocon could serve both functions. Tim44 serves to bridge the interactions between Tim23/17 and both Hsp70 and J-protein Pam18 (via Pam16). In addition, binding of substrate by Hsp70 destabilizes its interaction with Tim44 [73, 86], thereby allowing binding of another Hsp70, and continuation of translocation (Fig. 6). For the motor to function efficiently, Hsp70 at the channel must be in the ATP-, not ADP-, bound state, to initiate interaction with the incoming polypeptide rapidly. Premature stimulation of ATP hydrolysis by the J-protein Pam18 in the absence of substrate (i.e., the translocating polypeptide) could occur, decreasing motor efficiency. But on the other hand, efficient motor function also requires rapid J-domain action as soon as the translocating polypeptide enters the matrix.

Discussion on the issue of keeping Hsp70 primed, in the ATP-state, has centered around Tim44’s interactions with multiple binding partners and the Pam18–Pam16 heterodimer. The idea that Tim44 may play an important role was boosted by the findings that the intrinsically disordered NTD binds preprotein targeting sequences [66, 87] in addition to Pam16/18 and Hsp70. Many scaffolding proteins involved in signal transduction and regulation [88] are intrinsically disordered, having different conformations, depending upon which of their binding partners they are interacting with. The idea that such conformational changes play a role in regulating motor function became more intriguing with the finding that the site to which the targeting sequence binds overlaps with residues important for binding of Hsp70 and Tim23 [66] (Fig. 6a). This made it tempting to speculate that binding of the targeting sequence at this site, upon entrance into the matrix, induces a conformational change in Tim44 NTD that “activates” the motor. Perhaps conformational changes in Tim44 bring the Pam18 J-domain in close proximity to its binding site on Hsp70 [66, 89]. On the other hand, the Pam16–Pam18 interaction interface may be altered in some way. The idea that interaction of Pam18 with Pam16 may regulate Pam18’s ability to stimulate Hsp70’s ATPase activity stems from the observation that the Pam16–Pam18 heterodimer has on the order of 50% of the stimulatory ability of Pam18 alone [61, 90]. However, Pam18 variants having substitutions that reduce activity more than this support efficient mitochondrial import and robust cell growth [61]. Also, a Pam18–Pam16 heterodimer of J- and J-like domain-containing fragments was found to be inactive on stimulation of Hsp70’s ATPase activity [62]. While this inactivity could be indicative of a regulatory function, it could also be due to the absence of adjacent sequences shown to be important for forming an active complex [91]. Thus, although the Pam18–Pam16 interaction is central to motor function and Tim44 has characteristics consistent with regulatory roles, it remains unresolved how either facilitates maintenance of the motor in a state primed for action.

## Next directions

As described above, considerable progress has been made towards understanding the action of Hsp70s in protein translocation on both sides of membranes. Many questions remain, however. On the cytosolic side of the membranes, as results of more genome-wide ribosome profiling studies become available, a better picture of Hsp70 interactions with nascent chains will develop, allowing more directed studies to understand the importance of these interactions. Also, as new information about organelle targeting systems emerge, it will be interesting to see how generally Hsp70 functions on the cytosolic side. For example, do Hsp70s play a role in the recently identified SND targeting system to the ER that uses the SEC61 translocon [92]? Regarding import motor function, clearly more detailed knowledge is needed to gain a mechanistic understanding not only of how this molecular machine acts but also how such efficiency is obtained. Unfortunately, the mitochondrial inner membrane translocases have been difficult to purify and resistant to structural analysis. Hopefully, the rapid advancements occurring in structural biology and single molecule approaches that have recently provided insight into the Tom40 translocon and Hsp70s [93, 94] will soon be productive for the TIM23 translocon and PAM as well.
